# Effects of a non-pharmacological multicomponent intervention on delirium, sleep quality, and total sleep time among emergency intensive care unit patients in South Korea: a non-equivalent control group time-series study

**DOI:** 10.7586/jkbns.26.034

**Published:** 2026-08-27

**Authors:** Hye-Ji Kim, Mi-Kyoung Cho

**Affiliations:** Department of Nursing Science, College of Nursing, Chungbuk National University, Cheongju, Korea

**Keywords:** Intensive care units, Patient care bundles, Delirium, Sleep quality, Sleep duration

## Abstract

**Purpose:**

This study examined the effects of a non-pharmacological multicomponent intervention on delirium, sleep quality, and total sleep time in patients admitted to an emergency intensive care unit (EICU).

**Methods:**

A non-equivalent control group time-series design was used. Data were collected from the EICU of a tertiary hospital in South Korea between October 2025 and March 2026. A total of 48 patients were included, with 24 each in the experimental and control groups. The 2-day intervention comprised delirium prevention, sleep promotion, and intervention adherence management. Delirium was assessed using the Korean Nursing Delirium Screening Scale, sleep quality using the Korean version of the Richards-Campbell Sleep Questionnaire, and total sleep time was measured using a wearable device. Data were analyzed using generalized estimating equations, analysis of covariance (ANCOVA), and two-way repeated-measures ANCOVA using SPSS version 26.0 program.

**Results:**

Delirium scores were significantly lower in the experimental group than in the control group, with significant group (χ^2^ = 5.21, *p* = .022), time (χ^2^ = 12.86, *p* = .005), and group-by-time interaction effects (χ^2^ = 10.76, *p* = .013). Adjusted post-test sleep quality was significantly higher in the experimental group than in the control group (F = 16.15, *p* < .001, partial η^2^ = .26). Total sleep time showed significant main effects of group and time, whereas the group-by-time interaction was not significant (F = 2.61, *p* = .113, partial η^2^ = .05).

**Conclusion:**

The intervention helped prevent increases in delirium scores and improved sleep quality among EICU patients. This intervention may provide a feasible nursing strategy for critical care settings without the need for specialized equipment.

## INTRODUCTION

### 1. Background

The intensive care unit (ICU) is a specialized setting in which critically ill patients receive intensive care supported by advanced patient monitoring systems and life-sustaining medical equipment [1]. However, although ICU treatment provides patients with opportunities for survival through immediate medical interventions, it may also induce negative experiences, including the loss of human dignity associated with diaper use and physical restraints, and anxiety related to unfamiliar environmental factors such as continuous noise and lighting [2]. These experiences may lead to anxiety, depression, and post-traumatic stress disorder (PTSD) after discharge [3], thereby highlighting the need for the management of post-intensive care syndrome, which encompasses physical, cognitive, and psychological sequelae resulting from ICU treatment [4]. Among these factors, delirium is a major contributor to cognitive and psychological impairment after ICU care [5].

The incidence of delirium in the ICU has been reported to range from 41.3% to 65.9% [6,7]. Delirium is associated with prolonged ICU stay [8], cognitive decline after ICU discharge [9], and PTSD [5]. Despite these clinical consequences, delirium is considered a modifiable factor that can be addressed through nursing interventions, emphasizing the need for early detection and development of effective interventions [10].

The ICU environment causes sleep fragmentation and melatonin deficiency, ultimately leading to sleep deprivation. Sleep deprivation is associated with delirium, impaired immune function, decreased respiratory muscle strength, delayed recovery, and increased mortality [11]. Furthermore, sleep and circadian rhythm disturbances have been implicated in the development of delirium through alterations in melatonergic and other neurotransmitter systems [12,13]. The experience of delirium itself has been reported as a major risk factor for sleep disturbance [14], suggesting a bidirectional relationship between these variables. Accordingly, sleep should be considered as a key component in the prevention and management of delirium.

Reflecting the interrelationship between sleep and delirium, the Society of Critical Care Medicine incorporated sleep into an integrated management framework in the Pain, Agitation/Sedation, Delirium, Immobility, and Sleep Disruption (PADIS) guidelines and recommended non-pharmacological multicomponent interventions [15]. Single interventions have shown inconsistent effects; however, multicomponent interventions have demonstrated significant effectiveness in reducing the incidence of delirium [16]. Orientation enhancement, cognitive stimulation, and sleep hygiene have been suggested as major intervention components [17]. The combined application of sleep promotion and cognitive stimulation is an effective intervention strategy [18]. Family participation has been shown to be effective as part of multicomponent interventions [19]. These findings support the need for non-pharmacological multicomponent interventions that integrate orientation enhancement, cognitive stimulation, sleep hygiene, and family participation. In South Korea, studies have reported the effects of non-pharmacological multicomponent interventions on sleep improvement [20]; however, studies simultaneously examining delirium and sleep remain limited. Among ICU patients, reduced sleep quality and decreased total sleep time interact with delirium and form a sequential pathway. Based on the integrated management framework proposed in the PADIS guidelines, further research is needed to examine the effects of non-pharmacological multicomponent interventions that comprehensively address delirium, sleep quality, and total sleep time within this pathway.

### 2. Study aim

This study aimed to examine the effects of a non-pharmacological multicomponent intervention on delirium, sleep quality, and total sleep time in patients admitted to the emergency intensive care unit (EICU) and to provide an evidence-based nursing intervention strategy.

### 3. Hypotheses

The following hypotheses were proposed:

Hypothesis 1: The experimental group receiving the non-pharmacological multicomponent intervention will demonstrate lower delirium scores than the control group.

Hypothesis 2: The experimental group receiving the non-pharmacological multicomponent intervention will demonstrate higher sleep quality scores than the control group.

Hypothesis 3: The experimental group receiving the non-pharmacological multicomponent intervention will demonstrate longer total sleep time than the control group.

## METHODS

### 1. Study design

This study employed a non-equivalent control group time-series design to examine the effects of a non-pharmacological multicomponent intervention on delirium, sleep quality, and total sleep time in patients admitted to the EICU. Intervention allocation and statistical analyses were conducted at the individual level. This study was reported in accordance with the Transparent Reporting of Evaluations with Nonrandomized Designs (TREND) guideline.

### 2. Participants

The sample size was calculated using G*Power version 3.1.9.7 (Heinrich Heine University Düsseldorf, Düsseldorf, Germany). The effect size (Cohen’s d = 0.92) was estimated by applying the conversion formula, d = ln(odds ratio [OR]) ÷ 1.81 [21], to the odds ratio (OR = 0.19) reported in a previous study [22] that examined the effects of a multicomponent intervention on delirium among older ICU patients. Using a two-tailed significance level of .05 and a statistical power of .80, the required sample size was calculated as 20 participants per group. Considering a dropout rate of 30%, 28 participants were recruited for each group, resulting in a total of 56 participants. The inclusion criteria were as follows: (1) adults aged 18 years or older; (2) patients able to communicate; (3) patients with light sedation, defined as Richmond Agitation-Sedation Scale (RASS) scores ranging from −2 to +1 [15]; and (4) patients without neurologic disorders, including dementia and stroke. The exclusion criteria were as follows: (1) patients without a family caregiver available for participation in the family intervention; (2) patients admitted to isolation rooms; (3) patients with hearing or visual impairments that were not correctable with assistive devices; and (4) patients scheduled for transfer within three days of ICU admission.

### 3. Instruments

#### 1) Delirium

Delirium was measured using the Korean Nursing Delirium Screening Scale (Korean Nu-DESC), adapted for use in Korea by Kim et al. [23] based on the Nu-DESC developed by Gaudreau et al. [24]. This instrument consists of five items: disorientation, inappropriate behavior, inappropriate communication, illusions or hallucinations, and psychomotor retardation. Each item is scored as 0 (absent) or 1 (present), with total scores ranging from 0 to 5. A score ≥ 2 indicates delirium. Due to the fluctuating nature of delirium, assessments were conducted three times daily during the intervention period (excluding pre- and post-tests). The sensitivity and specificity of the Korean Nu-DESC were .81 and .97, respectively [23]; in this study, they were 1.00 and .91, respectively. These values were calculated by comparing concurrent Korean Nu-DESC and Confusion Assessment Method for the ICU ratings by the researcher and assigned nurse, respectively, across all participants and time points.

#### 2) Sleep quality

Sleep quality was measured using the Korean version of the Richards-Campbell Sleep Questionnaire (K-RCSQ), translated and validated by Kim et al. [25] based on the original RCSQ developed by Richards et al. [26]. The instrument consists of five visual analog scale items that assess sleep depth, sleep latency, sleep fragmentation, time to return to sleep, and overall sleep quality. Each item is rated from 0 (very poor) to 100 (excellent). The mean score represents overall sleep quality, while higher scores indicate better sleep quality. Cronbach’s α was .90 at development, .96 for the Korean version, and .95 in this study.

#### 3) Total sleep time

Total sleep time was measured using a Galaxy Fit 3 (Samsung Electronics Co., Ltd., Suwon, Korea). The device uses an accelerometer and optical heart rate sensor to monitor movement and heart rate during sleep, and classifies sleep into wakefulness, rapid eye movement (REM), light, and deep sleep stages. Total sleep time was defined as the sleep duration excluding wakefulness. Data were collected using wrist-worn devices and extracted using the Samsung Health application (Samsung Electronics Co., Ltd., Suwon, Korea). In a validation study of the Galaxy Watch 3 against polysomnography, total sleep time showed moderate agreement (intraclass correlation coefficient = .64) [27]. However, evidence regarding the clinical validity of the Galaxy Fit 3 remains limited; therefore, wearable devices were used as supplementary tools rather than as diagnostic instruments [28].

### 4. Intervention

#### 1) Development of non-pharmacological multicomponent intervention

The non-pharmacological multicomponent intervention was developed based on previous studies [19,22,29] to ensure evidence-based applicability. Content validity was assessed by an expert panel consisting of one nursing professor, one EICU head nurse, one intensivist, and one nurse with a master’s degree and over 10 years of clinical experience. Each item was evaluated using a 4-point Likert scale. Ratings of 1–2 were recoded as 0 (inappropriate) and those of 3–4 as 1 (appropriate) to calculate the item-content validity index (I-CVI). All items demonstrated an I-CVI of 1.0. Based on expert feedback, minor revisions and additions were made to finalize a clinically applicable intervention.

#### 2) Experimental group: Non-pharmacological multicomponent intervention

##### (1) Delirium prevention intervention

A cognitive and sensory stimulation-based delirium intervention was conducted based on previous studies [22,29]. Participants were provided with personal belongings and photographs of their families or friends. If used, glasses or hearing aids were placed at the bedside.

For orientation reinforcement on the first day of the intervention, the researcher explained the location of the EICU clock and attached a laminated card indicating the current date, admission date, and diagnosis to a bedside monitor. Two additional silent clocks were installed to complement the three existing clocks, ensuring the visibility of time information from all beds and minimizing blind spots. The assigned nurse assessed orientation at least every four hours and provided orientation cues regarding date, time, and place. For delirium monitoring, the researcher reviewed the nursing records and assessed delirium status three times daily (08:00, 16:00, 23:00). Regular delirium assessment using validated tools is essential for delirium prevention and management in ICU patients [29].

The family participation intervention involved playing approximately 120-second voice messages recorded by family members twice daily (10:00, 16:00). Earphones were applied during playback, and the volume was adjusted from a 50% baseline to a comfortable level for the participant. Messages were recorded during visits when possible or were obtained via telephone or messaging applications. The content included encouragement, explanation of ICU treatment, orientation information, and use of familiar language to enhance emotional connection [19].

##### (2) Sleep promotion intervention

Earplugs (white, 3M Co., Seoul, Korea) and sleep masks (Sonata Elegance Sleep Mask, PBK Trading Co., Goyang, Korea) were provided from 00:00 to 05:00 [22]. Participants who refused to use either device did not receive the intervention. For participants who refused to use sleep masks, environmental lighting was adjusted to improve their sleep conditions. Based on prior evidence that low nighttime illuminance levels (mean 11 ± 9 lux [lx]) do not adversely affect sleep quality or duration in ICU patients [30], environmental lighting was maintained at ≤ 20 lx during the intervention period (00:00–05:00). Two overhead bed lights were turned off and illuminance was measured approximately 50 cm above the participant’s head to ensure that the lighting level remained at or below 20 lx. A non-contact thermometer was used to minimize sensory stimulation during sleep.

##### (3) Intervention adherence management

To ensure standardization and adherence, a checklist for the multicomponent intervention was attached to the monitor at each experimental bed. The assigned nurse recorded the completion of each intervention item, and the researcher reviewed the checklist daily to monitor adherence.

#### 3) Control group: Usual nursing care

The control group received the usual nursing care for two days. At shift changes (08:00, 16:00, 23:00), the assigned nurse greeted and introduced themselves to participants, assessed their consciousness and sedation levels using the RASS, and evaluated their orientation. Vital signs and hemodynamic status were routinely monitored and basic nursing care and assessments were provided and conducted. At night, environmental lighting was minimized and a quiet environment was maintained as much as possible. Family visits were permitted as per hospital policy. Additional visits were allowed upon admission or when patients were clinically indicated.

### 5. Data collection

Prior to data collection, the study purpose and procedures were explained to the ICU attending physician, nursing department, EICU head nurse, and EICU nurses at Chungbuk National University Hospital to obtain their cooperation. Data were collected from patients admitted to the EICU from October 1, 2025, to March 16, 2026. The EICU has an open unit structure; therefore, the simultaneous recruitment of the control and experimental groups could have led to intervention contamination. To minimize this risk, a non-equivalent control group time-series design was used. Participants were sequentially assigned according to their admission order. To reduce the selection bias associated with non-random assignment, identical inclusion and exclusion criteria were applied to both groups, and baseline homogeneity was tested. The possibility of maturation effects due to the different data collection periods between the groups was considered; however, no significant changes occurred in the EICU’s operational environment or nursing care system during the study period.

Blinding was not feasible because of the non-randomized design. Participants were not informed of the group allocation, which limited their awareness of the assignment. Intervention providers were aware of the group allocation due to the nature of care delivery; however, standardized intervention protocols and checklists were used to minimize performance bias. The outcome assessor (researcher) was not blinded to the group allocation; therefore, standardized measurement tools and structured procedures were used to ensure consistency in data collection.

The intervention was delivered by assigned nurses. Prior to implementation, the researcher provided training based on the non-pharmacological multicomponent intervention protocol and checklist. To ensure intervention fidelity, a checklist was placed at each participant’s bedside and nurses recorded the completion of each intervention component. The researcher reviewed the checklist daily to monitor adherence and maintain consistency in intervention delivery.

#### 1) Pre-test

The study was conducted among patients admitted to the EICU who met the eligibility criteria and provided written informed consent. On the day of enrollment (D0), the researcher assessed baseline delirium using the Korean Nu-DESC and reviewed participants’ medical records to obtain general and clinical characteristics. To assess the baseline total sleep time, a Galaxy Fit 3 was applied to the participant’s right wrist from 00:00 to 05:00 on the day following enrollment (D1) by the assigned nurse or researcher. If application to the right wrist was not feasible, the device was applied to the left wrist. The researcher verified the total sleep time. At 07:00 on D1, the K-RCSQ was administered to assess baseline sleep quality.

#### 2) Intervention

Beginning at 07:00 on D1, the control group received usual nursing care, and the experimental group received a non-pharmacological multicomponent intervention for two days. During the intervention period, delirium screening scores were assessed thrice daily (08:00, 16:00, 23:00) through medical record reviews, and the highest daily score was used for the analysis. From 00:00 to 05:00 on the second (D2) and third (D3) days following enrollment, a Galaxy Fit 3 was applied to the participants' wrists by the assigned nurse or researcher. Total sleep time measured on D2 was considered the mid-intervention value, and total sleep time measured on D3 was defined as the post-intervention value; both values were verified by the researcher.

#### 3) Post-test

**Figure 1.**
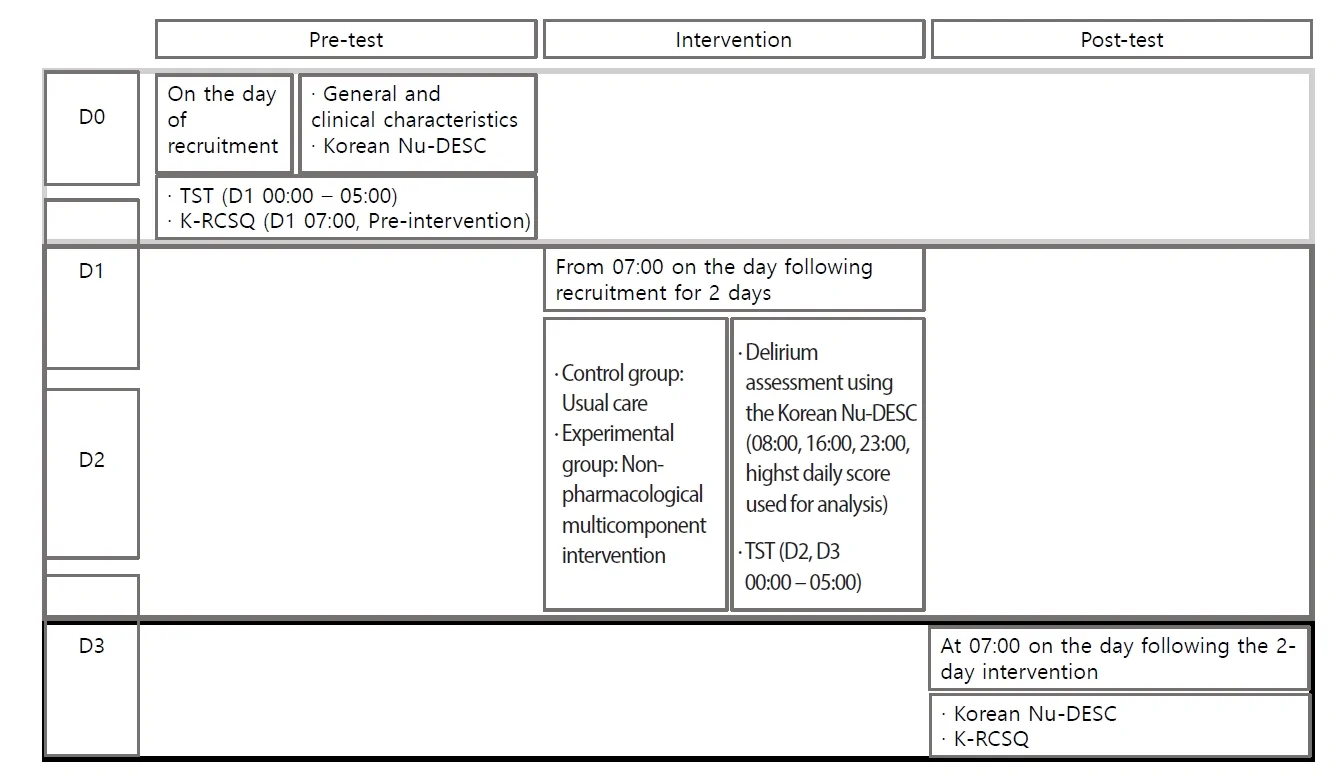
Data collection procedure. Korean Nu-DESC = Korean version of the Nursing Delirium Screening Scale; K-RCSQ = Korean version of the Richards-Campbell Sleep Questionnaire; TST = Total sleep time, measured from 00:00 to 05:00 on D2 and D3, with measurement completed at 05:00; D0 = Day of participant recruitment; D1 = First day of intervention; D2 = Second day of intervention; D3 = Day of post-intervention assessment (following completion of the 2-day intervention).

At 07:00 on D3, following completion of the 2-day intervention, the K-RCSQ was administered to assess post-intervention sleep quality. After questionnaire completion, post-intervention delirium screening scores were assessed using the Korean Nu-DESC (Figure 1).

### 6. Data analysis

No missing data were identified. Data from the 48 participants who completed the study (24 each in the control and experimental groups) were analyzed using SPSS version 26.0 (IBM Corp., Armonk, NY, USA). General and clinical characteristics were analyzed using descriptive statistics, and group homogeneity was tested using the Mann–Whitney U, independent t-test, and *χ*^2^ test. The normality of the dependent variables was assessed using the Shapiro–Wilk test. The baseline homogeneity of the dependent variables was examined according to normality test results. The Mann–Whitney U test was used for delirium scores because normality was not satisfied, whereas independent t-tests were used for sleep quality and total sleep time. Delirium screening scores were analyzed using generalized estimating equations (GEE) with a normal distribution, identity link function, exchangeable working correlation structure, and robust standard errors to assess group, time, and group-by-time interaction effects. As baseline differences may influence the estimation of intervention effects, baseline values of sleep quality and total sleep time were included as covariates. Sleep quality was analyzed using an analysis of covariance (ANCOVA), and total sleep time was analyzed using a two-way repeated-measures ANCOVA, with D2 and D3 as repeated measurement time points. Statistical significance was set at *p* < .05.

### 7. Ethical considerations

This study was approved by the Institutional Review Board (IRB) of a university prior to data collection (IRB No. CBNU-2025-A-0087). Permission to use the Korean Nu-DESC and K-RCSQ was obtained from their respective authors via email. Participants were informed of the study purpose and procedures, protection of personal information, their right to withdraw at any time, and the absence of any disadvantages associated with withdrawal. After receiving a full explanation of the study, the participants who voluntarily agreed to participate provided written informed consent. No participant requested withdrawal during the intervention period, and no adverse events related to the use of earplugs or sleep masks were reported. The collected data were used solely for research purposes, coded to ensure confidentiality, and stored in a secure location with restricted access. The research files will be permanently destroyed after the required retention period. If the effectiveness of the intervention is demonstrated, the same intervention will be offered to the control group upon request after study completion. Participants received a small gift (a sleep mask and earplugs; approximately KRW 5,000) in appreciation for their participation.

## RESULTS

### 1. Participant flow

Control group participants (n = 28) were recruited sequentially from October 1 to December 20, 2025, and the intervention procedures were completed. Subsequently, experimental group participants (n = 28) were recruited sequentially from January 1 to March 16, 2026. In the control group, four participants were lost to follow-up due to an inability to communicate following acute stroke (n = 1) and intra-hospital transfer to another unit (n = 3). In the experimental group, four participants were lost to follow-up due to inability to communicate related to clinical deterioration (n = 2) and intra-hospital transfer to another unit (n = 2).

**Figure 2.**
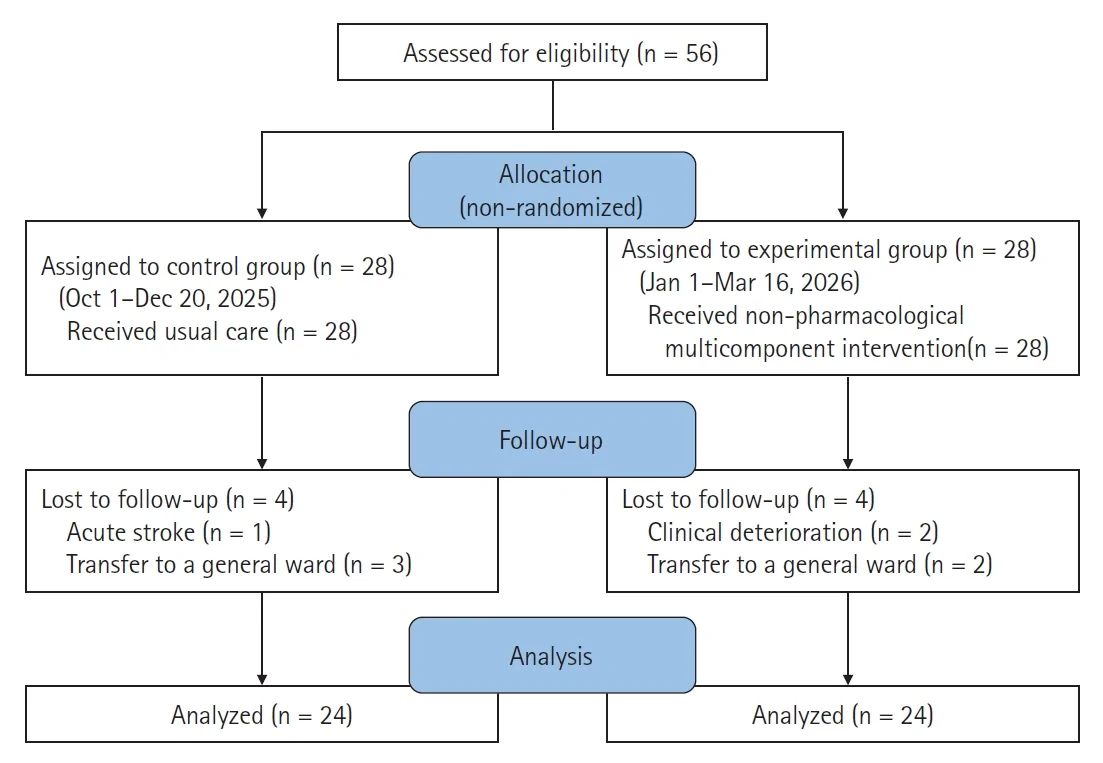
Flow diagram of the study showing non-randomized sequential group assignment.

A per-protocol analysis was then conducted. The eight participants lost to follow-up did not complete the intervention for clinical reasons; therefore, a comparison of baseline characteristics between the participants lost to follow-up and the analyzed participants was not performed. Finally, 24 participants from each of the control and experimental groups (N = 48) were included in the analysis (Figure 2).

### 2. Baseline homogeneity

Homogeneity testing of the general and clinical characteristics and main variables showed no significant differences between groups, confirming baseline homogeneity (Table 1).

### 3. Hypothesis testing

GEE showed significant group (*χ*^2^ = 5.21, *p* = .022), time (*χ*^2^ = 12.86, *p* = .005), and group-by-time interaction effects (*χ*^2^ = 10.76, *p* = .013). Therefore, Hypothesis 1 was supported (Table 2). Pre-test sleep quality was significantly associated with post-test sleep quality in both the experimental (r = .70, *p* < .001) and control groups (r = .73, *p* < .001); therefore, baseline sleep quality was included as a covariate. Prior to analysis, assumptions of homogeneity of variance, homogeneity of regression slopes, normality, and absence of extreme outliers were examined and met. An ANCOVA was conducted to compare post-test sleep quality between groups after adjusting for baseline values. The covariate effect was significant (F = 46.39, *p* < .001). Post-test sleep quality differed significantly between groups (F = 16.15, *p* < .001, $\eta_{p}^{2}$ = .26), with higher adjusted scores in the experimental group than in the control group. Therefore, Hypothesis 2 was supported (Table 3). The pre-test total sleep time was significantly correlated with the post-test total sleep time (r = .34, *p* = .017) and was included as a covariate. Prior to analysis, assumptions of homogeneity of regression slopes, homogeneity of covariance matrices, normality, and absence of extreme outliers were examined. Although the Shapiro–Wilk test indicated a deviation from normality in the experimental group at D3 (W = .90, *p* = .022), the Q–Q plots suggested an approximately normal distribution; therefore, the assumption of normality was considered acceptable. A two-way repeated-measures ANCOVA was conducted with D2 and D3 as within-subject factors. The main effects of group (F = 7.40, *p* = .009, $\eta_{p}^{2}$ = .14) and time (F = 4.98, *p* = .031, $\eta_{p}^{2}$ = .10) were significant, whereas the group-by-time interaction was not significant (F = 2.61, *p* = .113, $\eta_{p}^{2}$ = .05). Therefore, Hypothesis 3 was not supported (Table 3).

## DISCUSSION

This study examined the effects of a non-pharmacological multicomponent intervention on delirium, sleep quality, and total sleep time in critically ill patients. GEE analysis showed significant group, time, and group-by-time interaction effects on delirium screening scores, indicating that changes in delirium screening scores over time differed between the groups. The experimental group maintained delirium screening scores at baseline throughout the study period, whereas the control group showed higher post-test scores than baseline, indicating different temporal patterns between the groups. This intervention may have helped prevent an increase in delirium symptoms over time, consistent with the results of previous studies [22,31]. Kang and Choi’s [31] systematic review primarily included mixed medical-surgical ICU populations, and Hwang and Kim [22] targeted patients aged 65 years and older in general ICUs, indicating differences in participant characteristics and ICU settings compared with the present study. Despite these differences, the consistency of the findings suggests that non-pharmacological multicomponent interventions may have stable effects on delirium reduction across different ICU settings.

This study’s intervention was developed based on the PADIS guidelines [15] and integrated sleep promotion, cognitive stimulation, and family involvement. The PADIS guidelines recommend multicomponent non-pharmacologic strategies over pharmacologic agents as the primary evidence-based approach to delirium prevention [15]. Sleep promotion was achieved by reducing nighttime light exposure and providing sleep aids to improve the sleep environment. Kang et al. [32] reported that shorter sleep duration and an increased wake after sleep onset ratio during the first night in the ICU were associated with a higher risk of delirium on the following day, supporting the link between sleep–wake cycle disruption and delirium development. This association may be attributable to circadian disruption and melatonin deficiency, which are known to contribute to neurotransmitter imbalance and subsequent delirium [12,13]. Therefore, the sleep intervention in this study may have helped reduce these risks. Orientation reinforcement, a central component of the PADIS guidelines rather than an adjunctive measure, directly addresses disorientation—a core diagnostic feature of delirium [24]. In addition, cognitive stimulation and emotional support, including orientation cues and family voice messages, may reduce the risk of delirium by alleviating confusion and anxiety [13]. Family involvement, in particular, has been associated with a lower risk of delirium, providing both emotional support and familiar orientation cues [33]. This mechanism is further supported by a recent multicenter randomized controlled trial, which demonstrated that scripted family voice recordings increased delirium-free days among mechanically ventilated patients, with a dose–response relationship between playback frequency and effect [34]. Collectively, these intervention components may have contributed to preventing the increase in delirium scores observed over time.

Delirium is a multifactorial condition resulting from the interaction of various risk factors. Previous studies have shown that non-pharmacological multicomponent interventions significantly reduce the occurrence of delirium [17] and that the combined application of sleep promotion and cognitive stimulation is among the most effective intervention strategies [18]. Therefore, this study’s intervention may have influenced changes in delirium scores by simultaneously addressing multiple risk factors. These findings support the clinical usefulness of non-pharmacological nursing interventions targeting biological, cognitive, and environmental risk factors underlying delirium.

Most participants in this study had Korean Nu-DESC scores below the cut-off value, indicating that delirium-positive cases were relatively uncommon. This may be explained by the exclusion of patients with neurological disorders who are at high risk for delirium. However, delirium scores in the control group gradually increased over time, suggesting the possibility of subsyndromal delirium (SSD). SSD is associated with increased long-term mortality and a higher risk of progression to delirium, highlighting the importance of early identification and preventive intervention [35,36].

This study is meaningful because it focused on patients admitted to the ICU through the emergency department (ED), whereas previous studies on delirium interventions primarily targeted medical, surgical, or postoperative ICU populations. ED admission has been identified as an independent risk factor for delirium [37]. However, evidence regarding non-pharmacological multicomponent interventions for patients admitted through the ED remains limited. Therefore, this study demonstrates the clinical feasibility and potential benefits of this intervention in an EICU setting.

After adjusting for baseline sleep quality, post-test sleep quality scores were significantly higher in the experimental group than in the control group. Although the control group had higher baseline sleep quality scores, the experimental group demonstrated higher adjusted post-test scores, suggesting a beneficial effect of the intervention. Sleep disturbances in the ICU arise from the combined effects of individual, environmental, and clinical factors [14]. To address these issues, earplugs and eye masks were used to reduce exposure to noise and light. When eye mask use was not feasible, room illumination was maintained below 20 lx to minimize environmental sleep disruption. Previous studies have suggested environmental modification as a key non-pharmacological strategy for promoting sleep [32], and significant improvements in RCSQ scores have been reported following the use of earplugs and eye masks [38]. Koçak and Arslan’s [38] study was conducted in a neurological ICU, whereas the present study was conducted in an emergency ICU. Despite the differences in patient characteristics and ICU settings, the findings were consistent. A meta-analysis by Karimi et al. [39] reported significant improvements in sleep quality following the same intervention. Collectively, these findings suggest that interventions targeting the sleep environment may improve sleep quality in various ICU settings. Modifications to nursing practices may also have contributed to reducing nighttime disturbances and improving the sleep environment [40].

Family participation in the intervention was implemented through the playback of prerecorded voice messages twice daily (10:00, 16:00). Anxiety is a well-known sleep disruption factor in critically ill patients [14]. Family voice interventions may reduce anxiety and cognitive disorientation through familiar auditory stimulation, thereby contributing to delirium reduction [41]. The emotional reassurance provided by this intervention may have contributed to the improvement in sleep quality observed in the present study. Therefore, the observed effects are likely attributable to the combined influence of environmental modification, emotional support, and reduced nighttime stimulation.

For total sleep time, while the group and time main effects were significant, the group-by-time interaction effect was not. Given the substantial inter-individual variability commonly observed among ICU patients, the present sample size and 2-day intervention period may have been insufficient to detect a group-by-time interaction effect. This finding is consistent with that of Knauert et al. [40], who reported that short observation periods and inter-study heterogeneity are key limitations in ICU sleep research. Furthermore, accelerometer-based devices may have reduced accuracy in detecting sleep in ICU patients under sedation or with limited physical movement [40]. To address this limitation, the K-RCSQ was administered in parallel. These methodological limitations should be considered when interpreting the findings.

Similarly, a previous study using earplugs and eye masks for five days reported no significant between-group differences in total sleep time [42]. In contrast, studies incorporating additional relaxation interventions demonstrated improvements in total sleep time, even over relatively short intervention periods [20]. These findings suggest that differences in intervention duration and components may influence the outcomes. Taken together, the absence of a significant interaction effect in the present study may be explained by the combined influence of the short intervention period, limited sample size, measurement constraints, and clinical characteristics of critically ill patients. The gradual increase in the total sleep time observed in the experimental group provides preliminary evidence that a longer intervention duration may yield more pronounced effects. Although the interaction effect for total sleep time was not significant, sleep quality improved significantly after adjusting for baseline values, suggesting that the intervention had a more detectable effect on sleep quality than on total sleep time. Because ICU total sleep time is often constrained by clinical care activities, improved sleep quality without a corresponding increase in duration may still be clinically meaningful.

The non-pharmacological multicomponent intervention was implemented according to the planned protocol; its overall feasibility was demonstrated. Interventions such as earplug and sleep mask application, illuminance control, and orientation provision were performed by nurses without the need for specialized equipment; no adverse reactions were reported. No participants withdrew voluntarily; all dropouts resulted from changes in clinical status. These findings suggest that this intervention can be implemented in a relatively stable and feasible manner in clinical practice. However, in the ICU setting, the implementation of certain intervention components may be limited, depending on patients’ clinical condition or level of sedation. Because this intervention relies on family participation, its applicability may be limited without an available caregiver; the use of earplugs and sleep masks may also incur additional costs. In addition, the researcher was not blinded to group allocation and participated in the assessment of delirium outcomes. Because of the nature of the intervention, the nurses providing the intervention could not be blinded and could infer group allocation during its implementation. Therefore, despite the use of standardized assessment tools and protocols, the possibility of observer and performance bias cannot be fully excluded. Future studies should explore implementation strategies that account for various clinical situations.

## CONCLUSION

This study examined the effects of a non-pharmacological multicomponent intervention on delirium, sleep quality, and total sleep time in patients admitted to an EICU. The experimental group showed lower delirium scores and higher sleep quality than the control group. For total sleep time, significant main effects of group and time were observed; however, the group-by-time interaction was not significant. These findings suggest the clinical applicability of this intervention as a non-pharmacological nursing strategy.

As this study was conducted using a single-institution, non-randomized design, further validation through multicenter, randomized controlled trials is required. Future studies should include a stricter control of confounding variables and longer intervention periods. Nursing education and standardized protocol development are also required to support wider clinical implementation.

## Figures and Tables

**Table 1.** Baseline Homogeneity of General, Clinical Characteristics, and Main Outcome Variables between the Two Groups (N = 48)

Characteristics	Categories	Exp. (n = 24)	Cont. (n = 24)	*χ*^2^, t, or Z	*p*
Age (years)		70.96 ± 11.20	68.75 ± 12.57	0.64	.524
Sex^✝^	Men	14 (58.3)	12 (50.0)	0.34	.562
	Women	10 (41.7)	12 (50.0)		
SAPS III score		46.29 ± 13.85	44.58 ± 11.87	0.46	.648
GCS score^‡^		14.33 ± 0.82	14.50 ± 0.88	−1.02	.306
Mechanical ventilation duration (days)^‡^		0.67 ± 1.13	1.13 ± 2.47	−0.13	.898
Number of restraints^‡^		0.42 ± 0.83	0.33 ± 0.76	−0.37	.714
Number of sedative medications^‡^		0.33 ± 0.70	0.42 ± 0.72	−0.57	.568
Delirium score^‡^		0.04 ± 0.20	0.04 ± 0.20	0.00	1.000
Sleep quality		47.83 ± 28.71	60.25 ± 26.50	−1.56	.126
Total sleep time (minutes)		159.46 ± 64.27	193.67 ± 63.70	−1.85	.070

Values are presented as n (%) or mean ± standard deviation. Exp. = Experimental group; Cont. = Control group; SAPS III = Simplified Acute Physiology Score III; GCS = Glasgow Coma Scale.

✝ Chi-square test.

‡ Mann-Whitney U test.

**Table 2.** Comparison of Delirium Score between the Two Groups (N = 48)

Variables	Exp. (n = 24)	Cont. (n = 24)	Source	*χ* ^2^	*p*
Pre-test	0.04 ± 0.20	0.04 ± 0.20			
D1	0.17 ± 0.38	0.33 ± 0.70	Group	5.21	.022
D2	0.17 ± 0.48	0.54 ± 0.83	Time	12.86	.005
Post-test	0.04 ± 0.20	0.46 ± 0.59	Time × Group	10.76	.013

Values are presented as mean ± standard deviation. Pre-test = assessment on D0 before the intervention; D1 and D2 = highest daily Korean Nu-DESC scores among three assessments; Post-test = assessment at 07:00 on D3 after completion of the intervention. Exp. = Experimental group; Cont. = Control group.

**Table 3.** Comparison of Sleep Quality and Total Sleep Time between the Two Groups (N = 48)

Variables		Exp. (n = 24)		Cont. (n = 24)		Source	F	*p*	$\eta_{p}^{2}$	95% CI
		M ± SD	Adj. M ± SE	M ± SD	Adj. M ± SE					
Sleep Quality	Pre-test	47.83 ± 28.71		60.25 ± 26.50			16.15	<.001	.26	[.07, .44]
	Post-test	64.42 ± 29.96	69.51 ± 4.66	47.75 ± 33.52	42.66 ± 4.66					
Total Sleep Time (minutes)	D2	171.29 ± 63.49	173.60 ± 15.83	148.04 ± 86.33	145.74 ± 15.30	Group	7.40	.009	.14	[.01, .32]
	D3	181.71 ± 86.52	192.42 ± 16.61	129.71 ± 90.63	119.00 ± 16.61	Time	4.98	.031	.10	[.00, .28]
						Time × Group	2.61	.113	.05	[.00, .22]

Exp. = Experimental group; Cont. = Control group; M = Mean; SD = Standard deviation; Adj. M = Adjusted mean (sleep quality covariate: pre-test sleep quality score; total sleep time covariate: pre-test total sleep time); SE = Standard error; $\eta_{p}^{2}$ = Partial eta squared; 95% CI = 95% confidence interval for partial η²; CI = Confidence interval; D2 = Second day of intervention; D3 = Day of post-intervention assessment (following completion of the 2-day intervention); Total sleep time was measured from 00:00 to 05:00 on D2 and D3, with measurement completed at 05:00 (see Figure 1). For all F tests, df = 1, 45.
